# Reconceptualizing mind wandering from a switching perspective

**DOI:** 10.1007/s00426-022-01676-w

**Published:** 2022-03-29

**Authors:** Yi-Sheng Wong, Adrian R. Willoughby, Liana Machado

**Affiliations:** 1 Department of Psychology and Brain Health Research Centre, University of Otago, William James Building, 275 Leith Walk, Dunedin, 9016 New Zealand; 2 Brain Research New Zealand, Auckland, New Zealand; 3 School of Psychology and Clinical Language Sciences, University of Reading Malaysia, Nusajaya, Malaysia; 4 Department of Psychology, Monash University Malaysia, Subang Jaya, Malaysia

## Abstract

Mind wandering is a universal phenomenon in which our attention shifts away from the task at hand toward task**-**unrelated thoughts. Despite it inherently involving a shift in mental set, little is known about the role of cognitive flexibility in mind wandering. In this article we consider the potential of cognitive flexibility as a mechanism for mediating and/or regulating the occurrence of mind wandering. Our review begins with a brief introduction to the prominent theories of mind wandering—the executive failure hypothesis, the decoupling hypothesis, the process**-**occurrence framework, and the resource**-**control account of sustained attention. Then, after discussing their respective merits and weaknesses, we put forward a new perspective of mind wandering focused on cognitive flexibility, which provides an account more in line with the data to date, including why older populations experience a reduction in mind wandering. After summarizing initial evidence prompting this new perspective, drawn from several mind**-**wandering and task**-**switching studies, we recommend avenues for future research aimed at further understanding the importance of cognitive flexibility in mind wandering.

## Introduction

In the past two decades, there has been growing interest in understanding the basic psychological processes of mind wandering and its underlying mechanisms (for a review, see Kvavilashvili & Rummel, 2020). Mind wandering refers to a phenomenon in which our attention shifts away from the task at hand toward task**-**unrelated thoughts (for reviews, see Smallwood et al., 2018; Smallwood & Schooler, 2006, 2015). It has been estimated that up to 50% of our waking time is spent mind wandering (Kane et al., 2007; Killingsworth & Gilbert, 2010). Despite its prevalence, most people view mind wandering from a negative perspective, in which our performance will drop if our mind wanders (for reviews, see Mooneyham & Schooler, 2013; Stan & Christoff, 2018). Indeed, a number of studies have found a negative association between mind wandering and primary task performance, including poorer performance in daily functioning (McVay et al., 2009) and driving (Baldwin et al., 2017; Yanko & Spalek, 2014). However, studies have also shown a positive relationship between mind wandering and both mood and cognition (e.g., Gable et al., 2019; Mazzoni, 2019; Welz et al., 2018). In order to understand how to minimize the costs of mind wandering and maximize its benefits, it is therefore important to determine what factors regulate its occurrence.

Despite mind wandering inherently involving a shift in mental set, no existing study to our knowledge has explicitly examined the role of cognitive flexibility in mind wandering. In the present article, we consider the potential of cognitive flexibility to help explain the nature of mind wandering and its tendencies. In an effort to advance the field, here we first briefly review and discuss the most prominent theories of mind wandering—the executive failure hypothesis (McVay & Kane, 2009, 2010, 2012b), the decoupling hypothesis (Smallwood & Schooler, 2006), the process**-**occurrence framework (Smallwood, 2013), and the resource**-**control account of sustained attention (Thomson et al., 2015). Then, we put forward a new perspective centered around cognitive flexibility that was prompted by findings from several mind**-**wandering studies in older adults (e.g., Gyurkovics et al., 2018; Jordão et al., 2019; Niedzwienska & Kvavilashvili, 2018) and mind**-**wandering studies involving task-switching paradigms in young adults (e.g., Arnau et al., 2020; Kam & Handy, 2014; Thomson et al., 2014). According to this new switching perspective, the reason why some populations (e.g., healthy older adults) experience distinct patterns of mind wandering stems from differences in cognitive flexibility, as instances of mind wandering are in fact instances of mental set shifting (see Murray & Krasich, 2020, for a similar argument). After presenting the evidence supporting this new perspective, we put forward recommendations for future research aimed at further understanding the importance of cognitive flexibility in mind wandering.

## Existing theories of mind wandering

### Executive failure hypothesis

According to the executive failure hypothesis (McVay & Kane, 2009, 2010, 2012b), the occurrence of mind wandering represents a failure in the control of executive resources to keep attention on the current task, as the suppression of mind wandering requires executive control. One key form of executive control is working memory. This hypothesis posits that individuals with lower working memory capacity (i.e., those who are less able to hold information in an active, quickly retrievable state; Engle, 2002) are less capable of maintaining task focus over extended periods of time and keeping mind wandering at bay, and consequently experience more mind wandering. In support of this hypothesis, studies have found that individuals with lower working memory capacity have higher self**-**reported mind**-**wandering rates than individuals with higher working memory capacity (McVay & Kane, 2009, 2012a, 2012b; Robison & Unsworth, 2018; Unsworth & Robison, 2020), and working memory capacity can reliably predict how often one’s mind wanders (Kane et al., 2001; Robison & Unsworth, 2018). A meta**-**analysis that examined the association between mind wandering, executive resources (e.g., working memory capacity), and task performance also provided support for this hypothesis, by showing that individuals with lower working memory capacity tend to engage in more mind wandering than individuals with higher working memory capacity (Randall et al., 2014).

Additional evidence in support of the executive failure hypothesis comes from research on mind wandering involving individuals with attention**-**deficit/hyperactivity disorder (ADHD; for a review, see Bozhilova et al., 2018)—a neurodevelopmental disorder characterized by inattentiveness, hyperactivity, and/or impulsivity (American Psychiatric Association, 2013). Using a probe**-**caught method, in which participants are intermittently interrupted during a vigilance task and probed to report where their attention is focused, Shaw & Giambra, (1993) showed that participants with a childhood history of ADHD diagnosis reported experiencing more task**-**unrelated thoughts during task performance than participants with no history of ADHD. Another study, which distinguished between deliberate mind wandering and spontaneous mind wandering, found that the occurrence of spontaneous, but not deliberate, mind wandering is positively associated with ADHD symptom severity (Seli et al., 2015). A similar result was obtained by Franklin et al., (2017), who found that a composite index of ADHD symptoms was positively correlated with both the frequency of mind wandering and a lack of awareness of mind wandering. More recently, Mowlem et al., (2019) demonstrated that elevated frequencies of mind wandering in adults with ADHD were positively correlated with self**-**reported measures of functional impairment across major life domains (e.g., school), and that the contribution of mind wandering to their impairment was independent of the core ADHD symptoms (inattention, hyperactivity, and impulsivity). Given that most individuals with ADHD have deficits in a variety of cognitive domains (e.g., Coutinho et al., 2018; Kasper et al., 2012; Ramos et al., 2020), these studies suggest that the excessive mind wandering they experience could be attributable to, at least in part, a failure of executive control (McVay & Kane, 2010).

### Decoupling hypothesis

The decoupling hypothesis (Smallwood & Schooler, 2006) suggests that decreased performance during mind wandering occurs primarily because our attention has become decoupled from the task at hand and is instead coupled to task**-**unrelated thoughts. This decoupling process is important as it prevents information processing of extraneous stimuli from interfering with our current mental focus (Smallwood et al., 2011) in order to ensure continuity of the train of thought (Smallwood, 2013). In other words, the decoupling hypothesis proposes that mind wandering is a process that relies on some of the same cognitive mechanisms involved in maintaining focused attention on the task at hand and thus directly competes with primary task performance for executive resources (Smallwood & Schooler, 2006).

Several event**-**related potential (ERP) studies have provided strong evidence for this hypothesis. For example, using the sustained attention to response task (SART; Robertson et al., 1997), Smallwood et al., (2008) showed that participants had a reduced P300 amplitude during self**-**reported mind wandering relative to on**-**task episodes. The P300 is a positive potential that peaks around 300 ms after stimulus presentation and is believed to reflect the extent to which the stimulus representation is updated in working memory (Donchin, 1981; Donchin & Coles, 1988) and/or the amount of executive resources allocated toward the stimulus (Kramer & Strayer, 1988; Wickens et al., 1983), with higher amplitude indicating more revision of the representations and/or more executive resources directed to processing the stimulus (for reviews, see Polich, 2007; Verleger, 2020). Because the P300 can provide an index of executive resources (e.g., Kramer & Strayer, 1988; Wickens et al., 1983), the decreased P300 amplitude during mind wandering indicates that our executive resources have been withdrawn, at least partly, from the primary task and are presumably directed toward task**-**unrelated thoughts (Smallwood, 2010; Smallwood & Schooler, 2006). Similar results were obtained in subsequent studies (Baldwin et al., 2017; Barron et al., 2011; Kam et al., 2014; Maillet et al., 2020).

### Process-occurrence framework

The process**-**occurrence framework, which was proposed by Smallwood, (2013), emphasizes the necessity of distinguishing between the onset of mind wandering and the continuation of the mind-wandering episode, linking McVay and Kane’s view (i.e., executive control failure) to the onset and Smallwood and Schooler’s view (i.e., mind wandering requires executive resources) to the continuation of the episode. According to this framework, under tasks requiring sustained attention, executive control can keep mind wandering at bay by ensuring the continuity of the train of task**-**related thought. However, when mind wandering occurs (e.g., due to executive control failure; McVay & Kane, 2009, 2010, 2012b), executive control shifts away (i.e., decouples) from the task at hand to enable the continuation of the mind-wandering episode (which consumes the same executive resources as the task at hand; Smallwood & Schooler, 2006), leaving insufficient executive resources for the primary task, thereby resulting in impaired task performance (Smallwood et al., 2012).

As proposed by Smallwood, (2013), there are at least two reasons why one would mind wander more. First, because the individual has difficulties in ensuring the continuity of their train of thought (see also McVay & Kane, 2010). This account could explain why individuals with ADHD tend to experience more mind wandering episodes (Bozhilova et al., 2018; Franklin et al., 2017; Mowlem et al., 2019; Seli et al., 2015; Shaw & Giambra, 1993). Second, because the individual considers their currently relevant personal concerns or unresolved goals (e.g., submit the assignment before the end of the day) as having higher priority than the demands of the task at hand, and thus shifts their attention toward these concerns (see also Klinger, 1975, 1999). This account could explain why older adults report less mind wandering than young adults (e.g., Jordão et al., 2019; Maillet & Schacter, 2016), as they tend to report having fewer concerns (Parks et al., 1989). In the former case, according to this framework (Smallwood, 2013), the individual should experience more *frequent* mind-wandering episodes, whereas in the latter case, the individual should experience *longer* episodes of mind wandering.

Although it is difficult to identify different states and processes involved in mind wandering, primarily because people normally do not realize when they first start mind wandering but only notice some time later (Smallwood, 2013; Zukosky & Wang, 2021), there has been one study to date that made an attempt at this (Voss et al., 2018). In this study, the researchers characterized the mind-wandering process by combining the self-caught and the probe-caught methods to estimate the duration of focus (defined as the time period from when the person first started focusing on the task at hand to the moment that mind wandering began), which was taken as a measure of one’s ability to maintain task focus and resist the occurrence of mind wandering, and the duration of mind wandering (defined as the time period from when the mind-wandering episode began to the moment that the individual caught themselves and reported it by pressing a button), which was taken as a measure of processes that keep one in the mind-wandering state. The researchers then investigated the association of these measures with working memory capacity. The results showed a strong positive correlation between the duration of focus and working memory capacity, which is consistent with previous findings that individuals with higher working memory capacity could maintain task focus over longer periods of time (Kane et al., 2001; McVay & Kane, 2009, 2012a, 2012b; Randall et al., 2014; Robison & Unsworth, 2018; Unsworth & Robison, 2020). However, no relationship was observed between the duration of mind wandering and working memory capacity, indicating that one’s tendency to engage in and detect the mind-wandering state was not affected by working memory capacity. The Voss et al., (2018) study, therefore, provides initial evidence to support the process**-**occurrence framework (Smallwood, 2013) that there are at least two distinct states and processes involved in mind wandering.

### Resource-control account of sustained attention

According to the resource**-**control account of sustained attention (Thomson et al., 2015), mind-wandering state is the default mental state and thus there is a continuous bias for executive resources to be directed toward mind wandering (see also Baird et al., 2011; Smallwood, 2013; Smallwood et al., 2009). This theory posits that the occurrence of mind wandering should be associated with decreases in motivation and/or effort to keep attention on the task at hand over time. In other words, as time-on-task increases, executive resources are less likely to be allocated to the task at hand and are more likely to be allocated to mind wandering, leaving insufficient executive resources for the primary task and thereby resulting in impaired task performance. In support of this theory, studies have found a negative association between time on task and primary task performance, and a positive association between time on task and rates of self**-**reported mind wandering (Brosowsky et al., 2020; Krimsky et al., 2017; McVay & Kane, 2012a; Thomson et al., 2014).

### Interim summary

Taken together, these four theories suggest that mind wandering is a state of decoupled information processing (Smallwood and Schooler’s view) that involves at least two component processes (Smallwood’s view): the initiation of mind wandering, which can be attributed to a failure of executive control (McVay and Kane’s view), and the continuation of the mind-wandering episode, which is a resource-dependent process (Smallwood and Schooler’s and Thomson et al.’s view). Although these four theories significantly advance our understanding of mind wandering, they are not without weaknesses or alternative interpretations. In the next section, we discuss these and put forward a new perspective of mind wandering focused on cognitive flexibility, which offers novel insight that aids towards our general understanding of mind wandering.

## Insight from a switching perspective

**Fig. 1:**
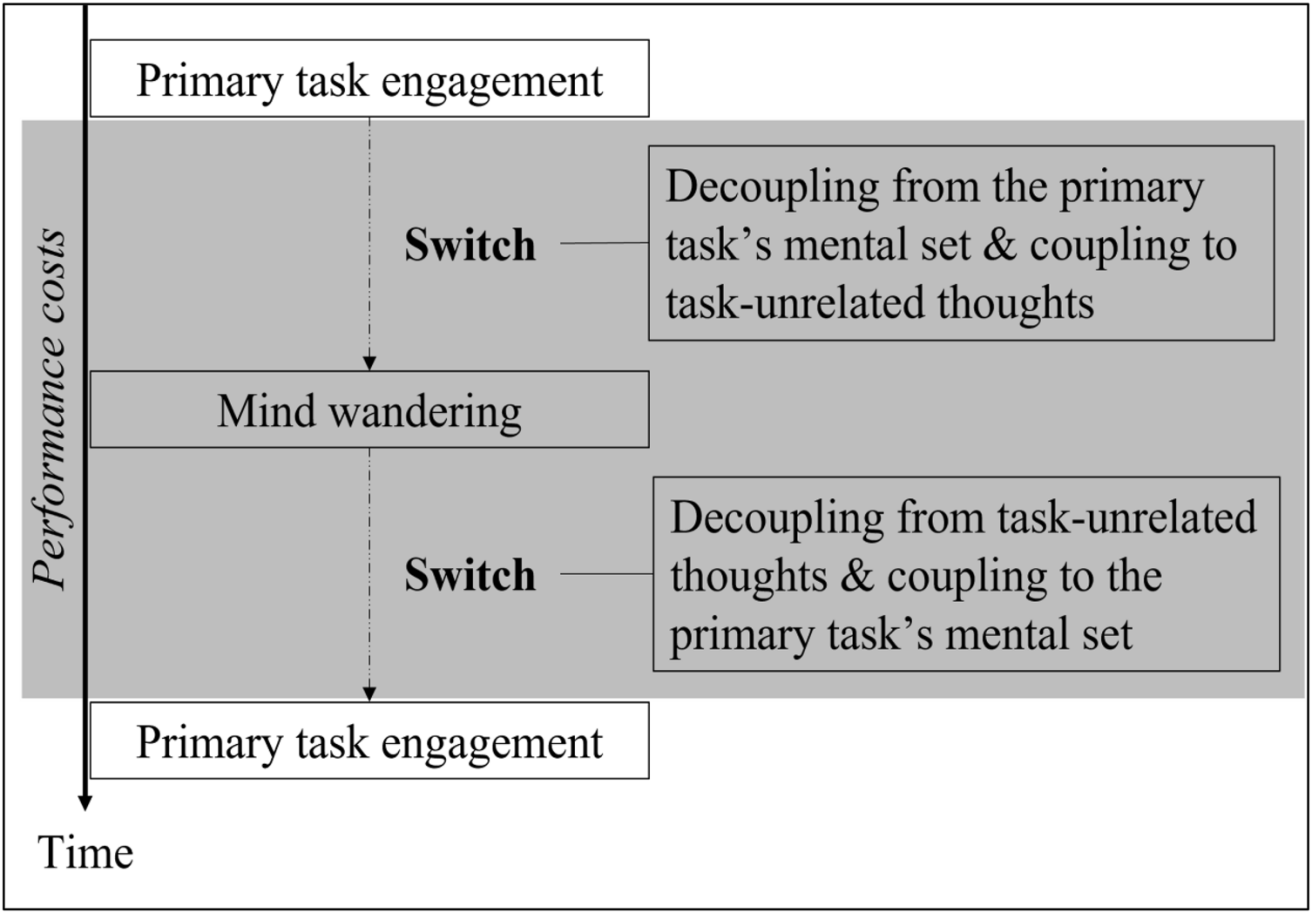
Conceptual framework for viewing mind wandering from a switching perspective. Maintenance of both primary task and mind**-**wandering mental sets occur in working memory. Each change of mental set requires inhibition of the previously relevant mental set. Grey area represents the time in which primary task performance costs arise

Cognitive flexibility, which can also be referred to as mental set shifting or switching, is one of the three core executive control functions (along with inhibitory control and working memory) that enables us to adjust our thoughts and actions in response to changed priorities or demands (Buttelmann & Karbach, 2017; Diamond, 2013; Miyake et al., 2000). To adapt to changing priorities, for example, we need to inhibit previously relevant thoughts and actions and activate newly relevant thoughts and actions in working memory. In this way, mental set shifting requires involvement of both inhibitory control and working memory (Diamond, 2013). With regard to mind wandering, we propose that it requires cognitive flexibility, as the occurrence of mind wandering entails inhibition of one’s primary task mental set (which enables decoupling to occur) and activation of task**-**unrelated thoughts in working memory (see Fig. 1).

Considering this switching view alongside existing frameworks and models such as the metacontrol state model (Hommel, 2015), which describes the balance between flexibility and persistence in cognitive processing (Zhang et al., 2020), may provide a way to understand variability in mind-wandering frequency. For instance, given that ADHD has long been found to be associated with dysregulated dopamine neurotransmission (Cook et al., 1995), and dopamine-related interindividual differences have been hypothesized to be associated with interindividual variability in metacontrol defaults (i.e., the control of the current cognitive-control settings; Hommel & Colzato, 2017), it seems possible that the increased mind wandering experienced by individuals with ADHD might be associated with a default metacontrol setting biased towards flexibility (i.e., weak goal shielding and weak mutual inhibition of task-related and task-unrelated mental sets) that may be related to imbalances of neurotransmitters. Moreover, given that older adults tend to report higher levels of positive affect (e.g., Frank et al., 2015) and motivation (e.g., Nicosia & Balota, 2021; Ryan & Campbell, 2021; Seli et al., 2021) than young adults during task performance, and situational factors such as these have been hypothesized to induce a metacontrol setting biased towards persistence (Hommel & Colzato, 2017), it seems possible that older adults’ less frequent reports of mind wandering (e.g., Arnicane et al., 2021; Jordão et al., 2019; Maillet & Schacter, 2016) might be associated with a stronger bias towards persistence (i.e., stronger goal shielding and stronger mutual inhibition of task-related and task-unrelated mental sets).

It may also be possible to account for the effect of mind wandering on creativity (e.g., Gable et al., 2019; Murray et al., 2021; Steindorf et al., 2021; Yamaoka & Yukawa, 2020) by integrating the switching perspective of mind wandering with both the metacontrol state model (Hommel, 2015) and the dynamic framework of thought (Christoff et al., 2016)—a model that provides insight into how thoughts that focus on personally or affectively salient information (i.e., automatic constraints) and thoughts that focus on goal-related information (i.e., deliberate constraints) dynamically influence the nature of thought over time. For example, given that both of these schemas emphasize the importance of flexibility or shifting between mental states in idea generation (for reviews, see Girn et al., 2020; Zhang et al., 2020), it seems possible that the relationship between mind wandering and creative thinking might be mediated by cognitive flexibility. This speculation may be worthy of future research.

In short, we believe that viewing mind wandering from a switching perspective may help explain, at least in part, why some populations experience more mind-wandering episodes while others experience fewer episodes, why participants who indicate higher levels of motivation are less likely to engage in mind wandering during task performance, and why mind wandering is sometimes linked to enhanced creativity.

### Limitations and an alternative viewpoint for the executive failure hypothesis

Although the executive failure hypothesis (McVay & Kane, 2009, 2010, 2012b), which posits that working memory plays a critical role in keeping mind wandering at bay, could explain the higher levels of mind wandering in healthy young adults with lower working memory capacity and individuals with ADHD, mind-wandering research involving other cohorts with lower working memory capacity has yielded results that challenge this account. For instance, a meta**-**analysis of 21 studies investigating aging effects in mind wandering revealed that older adults tend to report fewer instances of mind wandering when engaged in cognitive tasks (Jordão et al., 2019). This is puzzling given that according to the executive failure hypothesis, one would expect the rates of mind wandering to increase—not decrease—in older adults (for a review, see Maillet & Schacter, 2016), as executive control functions generally decline with advancing age (e.g., Craik & Salthouse, 2011; Foster et al., 2007; Machado, 2021). Furthermore, using the SART, Gyurkovics et al. (2018) found that individuals with early-stage Alzheimer’s disease reported experiencing fewer task**-**unrelated thoughts and more task**-**related thoughts than healthy age**-**matched controls, again indicating reduced incidence of mind wandering despite individuals with Alzheimer's disease showing declines in executive functioning (Guarino et al., 2018). Similar results have been reported in studies involving individuals with Parkinson’s disease (Walpola et al., 2020), who are also known to suffer from executive dysfunction (Flannery et al., 2018; McKinlay et al., 2010; Ramos & Machado, 2021), and individuals with amnestic mild cognitive impairment (Niedzwienska & Kvavilashvili, 2018).

A counterargument to the claim that lower levels of mind wandering in these older populations stand against the executive failure hypothesis stems from the fact that mind**-**wandering studies mostly rely on self**-**report measures. In relation to this, some researchers have attributed the finding of reduced mind wandering in healthy and cognitively impaired older adults to a lack of validity of their mind**-**wandering reports (Gyurkovics et al., 2018; Jackson & Balota, 2012; Zavagnin et al., 2014). However, several studies have demonstrated that these populations’ self**-**reported mind-wandering data are as valid as those by controls, by demonstrating that during self**-**reported off**-**task episodes, the two groups exhibited similar levels of disrupted task performance (e.g., Arnicane et al., 2021; McVay et al., 2013; Niedzwienska & Kvavilashvili, 2018). Moreover, eye movement (Frank et al., 2015) and brain activation (Maillet & Rajah, 2016; Walpola et al., 2020) patterns reliably predicted self**-**reported mind**-**wandering episodes in older adults and individuals with Parkinson’s disease, indicating that they were able to report their mind**-**wandering episodes accurately. These results, therefore, suggest that the explanation that decreased mind wandering relates to lack of validity of mind-wandering measures in these older populations does not hold up.

**Fig. 2:**
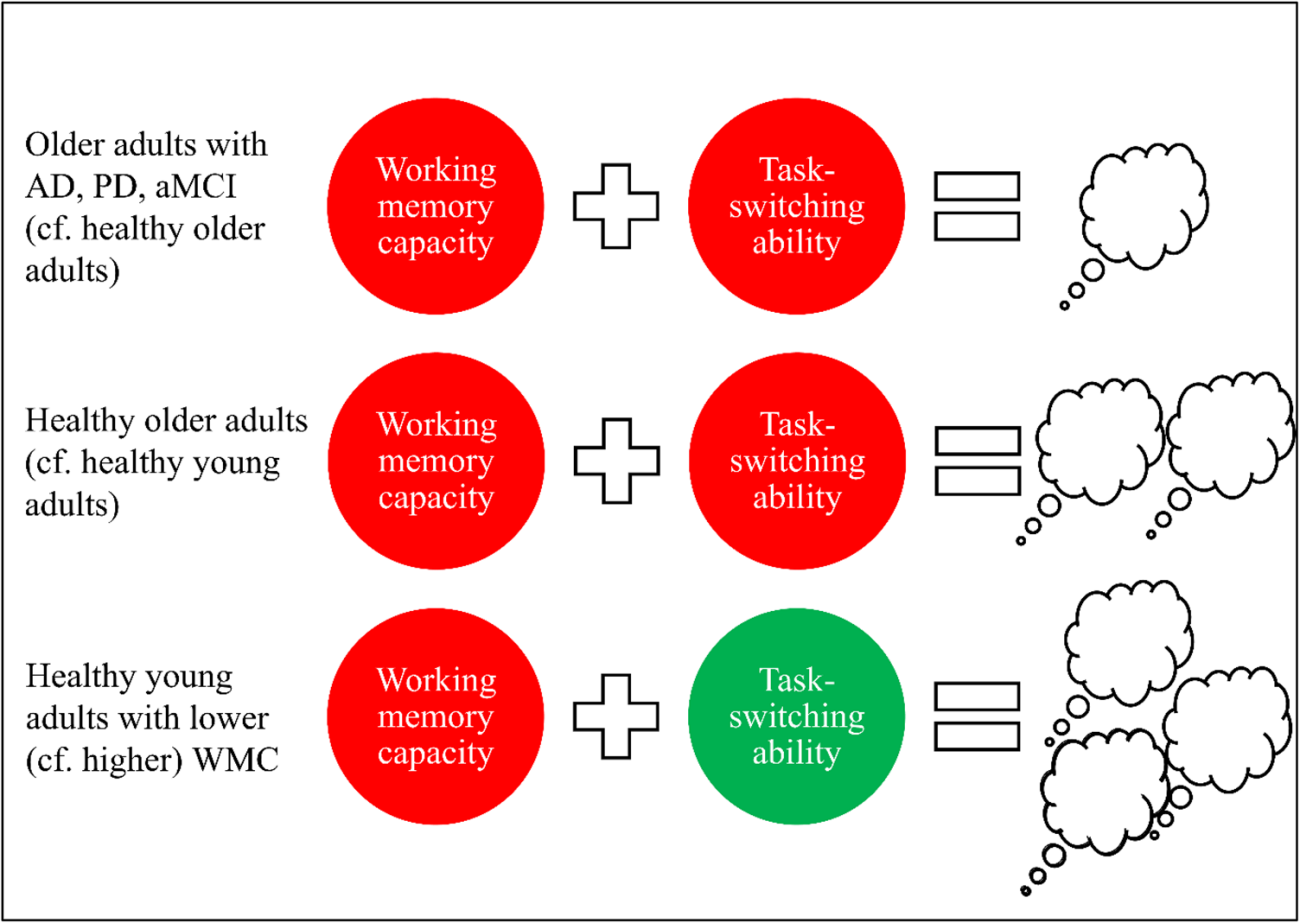
Summary of results gathered from several mind-wandering, working memory, and task-switching studies*.* *AD* Alzheimer’s disease, *PD* Parkinson’s disease, *aMCI* amnestic mild cognitive impairment, *WMC* working memory capacity. Red circle represents poorer performance and green circle represents better performance, relative to the comparison group. White thought bubble cloud represents mind-wandering frequency, with fewer clouds representing less frequent occurrences of mind wandering, relative to the comparison group

To shed light on decreased mind wandering in older cohorts, here we offer an alternative account of mind wandering focused on cognitive flexibility (for a summary of other alternative explanations for age-related declines in mind wandering, see Seli, Maillet, et al., 2017; Seli, Ralph, et al., 2017). According to this account, the reduced mind**-**wandering frequency seen in healthy older adults and those with Alzheimer’s disease, Parkinson’s disease, and amnestic mild cognitive impairment could be attributable, at least partly, to declines in cognitive flexibility (e.g., a reduced ability to switch between task**-**related and task**-**unrelated thoughts; see Fig. 2). This line of reasoning fits well with research showing that these older populations generally have a reduced capability to activate (e.g., to initiate a switch of mental set) and maintain cognitive representations (e.g., to maintain the new mental set; Craik & Salthouse, 2011; Lindenberger & Mayr, 2014; Traykov et al., 2007), and exhibit longer response times and/or higher error rates on switch trials relative to repetition trials (e.g., Brett & Machado, 2017; Rey-Mermet & Gade, 2018; Wasylyshyn et al., 2011).

Furthermore, given that previous research in healthy young adults has revealed a negative association between working memory capacity and mind**-**wandering frequency (McVay & Kane, 2009, 2012b; Robison & Unsworth, 2018; Unsworth & Robison, 2020), and a negative correlation between working memory capacity and task**-**switching performance (Miyake et al., 2000; Oberauer et al., 2003; Shipstead et al., 2015; for more details, see Draheim et al., 2016), it seems plausible that healthy young adults with lower working memory capacity might be more capable of adjusting their executive resources to different mental sets (including task-unrelated mental sets) due to their superior switching abilities (see the “Future directions” for further elaboration of this conjecture; see Fig. 2). In short, although the executive failure hypothesis (McVay & Kane, 2009, 2010, 2012b) holds up quite well for younger populations, the switching account of mind wandering put forward here could potentially also explain the patterns of results in these populations. Moreover, in contrast to the executive failure hypothesis which cannot account for the lower levels of mind wandering observed in older populations (as it predicts that these populations should exhibit elevated levels of mind wandering due to having lower working memory capacity), our switching account of mind wandering fits well with the existing data, suggesting that cognitive flexibility may play a more important role than working memory does in mediating and/or regulating the occurrence of mind wandering.

### An alternative interpretation for the decoupling hypothesis

Although studies using ERPs have provided evidence in favor of the decoupling hypothesis (Smallwood & Schooler, 2006) by demonstrating that mind wandering reduces the cortical processing of the task at hand (as reflected by the reduced P300 amplitude during mind wandering; Baldwin et al., 2017; Barron et al., 2011; Kam et al., 2014; Maillet et al., 2020; Smallwood et al., 2008), these findings could also be interpreted from a switching perspective. Previous research on task switching has consistently revealed a reduced P300 amplitude on switch trials relative to repetition trials (e.g., Barcelo et al., 2002; Hsieh & Cheng, 2006; Kieffaber & Hetrick, 2005; Mueller et al., 2007; Poljac & Yeung, 2014; Vandamme et al., 2010; Wylie et al., 2003). According to Jost et al. (2008), the reduced P300 amplitude in switch trials is thought to indicate that context updating (i.e., the comparison of the attributes of an incoming stimulus with an internal representation and the subsequent updating of the internal representation; Donchin, 1981; Donchin & Coles, 1988) is less easily achieved. Another explanation comes from Wylie et al., (2003), who suggested that the reduced P300 amplitude reflects a competition between task sets or rules, with the idea being that on switch trials the competition between task**-**specific response sets or rules is greater. This results in both a reduction in P300 amplitude and an increase in response time and/or error rate because the activation of the currently relevant task representation is less enhanced. Using the findings from these task**-**switching studies as a foundation, we posit that the attenuated P300 amplitude during mind wandering could reflect two possible processes, including (*a*) less efficient context updating in working memory (Donchin, 1981; Donchin & Coles, 1988), and/or (*b*) competition between primary-task representations and task**-**unrelated thoughts.

Building on this alternative perspective, we suggest that the decoupling process is a component of the switching process. As such, mind wandering could be considered as a subset of task switching that typically would run serially with task performance (i.e., serial multitasking; e.g., Huijser et al., 2018; for more details, see Taatgen et al., 2021), although a recent study has shown that mind wandering can also run parallelly with particular kinds of tasks (i.e., parallel multitasking; e.g., Brosowsky et al., 2021). Furthermore, in the context of serial processing of multiple mental sets, switch costs should be observed regardless of whether the shift is from external to internal (e.g., shifting from a SART to task-irrelevant personal concerns), internal to external (e.g., shifting from task-irrelevant personal concerns back to the SART), or internal to internal (e.g., shifting from mental arithmetic to task-irrelevant personal concerns). According to our view, the detrimental effect of mind wandering on primary task performance reflects the costs of switching between mental sets (i.e., decoupling from the primary task’s mental set and coupling to task**-**unrelated thoughts; see Fig. 1), in addition to the costs of not paying attention to the primary task while decoupled. This proposal is in line with research on task switching that demonstrated that switch costs could still be observed when the tasks were relatively simple, when the task sequence was predictable, and when there was a cue signaling the upcoming switch (Koch, 2003).

### A possible way to test the process-occurrence framework

Within the process-occurrence framework, Smallwood aptly noted the following:

…the processes that ensure the continuity of the experience of an internal train of thought are similar to those that can be engaged in standard task-relevant paradigms, and as a result, these processes are becoming reasonably well understood. By contrast, our understanding of why mind wandering occurs is less well specified, in part because we are unable to identify the moment of ignition for the state. (Smallwood, 2013, p. 532).

Indeed, as mentioned earlier, one major challenge in investigating the length of mind-wandering episodes is how to determine the “when” of mind wandering (Franklin et al., 2013). Although Voss et al., (2018) have provided evidence in favor of the process-occurrence framework by identifying different states and processes of mind wandering (see the “Process-occurrence framework” for more details), one key limitation of this study, as pointed out by the researchers themselves, was that their assessment methods hinged on the assumption that the only way for an individual to redirect their attention from mind wandering back to the task at hand is through a mechanism reliant upon self-awareness (i.e., the meta-awareness system; Schooler et al., 2011). If one can return to a task-focused state without relying on such a mechanism (e.g., decoupling from task-unrelated thoughts and coupling to the primary task’s mental set without conscious awareness), and can have multiple switches between task-focused and mind-wandering states during a single self-caught episode, then the estimated duration of focus and mind-wandering episodes (defined earlier in the “Process-occurrence framework”) could in fact reflect multiple focus-mind-wandering episodes, rather than the duration of each individual task-focused/mind-wandering state (Voss et al., 2018).

To shed light on how to measure the component processes of mind wandering while acknowledging that mind wandering often consumes executive resources that are needed to perform the task (i.e., parallel processing of both high cognitive demand tasks and mind wandering is difficult to achieve; Smallwood & Schooler, 2006; Thomson et al., 2015), here we put forward a new approach that links the onset of mind wandering to the onset of a new mental set, and the continuation of the mind-wandering episode to the continuation of the new mental set. Previous task-switching studies have identified a number of distinct cognitive processes underlying an attentional set switch (e.g., Meiran et al., 2000; Rushworth et al., 2002, 2005). For instance, Rushworth et al., (2002) found that mental set shifting consisted of at least three component cognitive processes, including: (*a*) initiation of a new mental set prior to selective focusing of attention, which was indexed by an early period of ERP modulation associated with dipole source estimates in the prefrontal cortex; (*b*) reconfiguration of the new mental set, which was indexed by a later period of ERP modulation associated with dipole source estimates at the ventromedial occipitotemporal junction; and (*c*) maintenance of the new mental set and possible interference from the previous mental set, which was indexed by the N200—a negative potential over the central posterior scalp that peaks around 200 ms after stimulus presentation and is believed to be associated with response suppression (Eimer, 1993; Kok, 1986; Patel & Azzam, 2005). Building on these findings, we posit that at the start of the mind-wandering episode, there should be activation in the prefrontal cortex (for a review, see Zamani et al., 2022)—a region that has been found to play a central role in cognitive flexibility (Dove et al., 2000; Miller & Buschman, 2013; Miller & Cohen, 2001; Sakai & Passingham, 2003; Sohn et al., 2000) and mind wandering (Bertossi & Ciaramelli, 2016; Burgess et al., 2007; Christoff et al., 2009; Fox et al., 2015; Stawarczyk & D’Argembeau, 2015).

Using these findings as a foundation, it seems possible that the “when” of mind wandering (i.e., the onset of mental set shifting) can be estimated, at least approximately, from activity in the prefrontal cortex measured prior to periods of self-reported mind wandering. This conjecture seems to fit with findings that non-invasive transcranial direct current stimulation of the prefrontal cortex can increase the propensity to mind wander (e.g., Axelrod et al., 2015, 2018; Filmer et al., 2019). To clarify, we postulate that positive-polarity stimulation “encourages” the recipient to initiate a switch of mental set, which according to the resource-control account of sustained attention (Thomson et al., 2015) is most likely to involve a switch to a task-unrelated mental set as mind wandering is thought to be the default mental state for most individuals.

### Limitations and a possible extension for the resource-control account of sustained attention

Although the resource-control account of sustained attention (Thomson et al., 2015), which suggests that the occurrence of mind wandering is associated with decreases in motivation and/or effort to keep attention on the task at hand over time, could explain why older adults tend to report fewer instances of mind wandering than young adults during cognitive task performance—either because they are more motivated to perform the primary task (Frank et al., 2015; Jackson & Balota, 2012; Seli et al., 2021; Seli, Maillet, et al., 2017; Seli, Ralph, et al., 2017) or because they have spent a larger proportion of their executive resources on the primary task (Craik & Byrd, 1982) and thus have fewer resources left over to exhibit mind wandering (Giambra, 1989; Krawietz et al., 2012; Maillet & Rajah, 2013)—this theory is not without its limitations. In particular, if executive control, which wanes over time on task, is required to prevent task-unrelated thoughts (i.e., the default mental state) from consuming executive resources needed for the task at hand, then given that healthy and cognitively impaired older adults generally have poorer executive control (e.g., Flannery et al., 2018; Guarino et al., 2018; McKinlay et al., 2010; Ramos & Machado, 2021), one might reasonably expect that as time-on-task increases, these older populations would report higher incidences of mind wandering and show more pronounced performance decrements. However, this prediction was not supported by Arnicane et al., (2021), who found that in comparison to the first block (i.e., the first 15 min) of a visual working memory task, in the sixth block healthy older adults reported similar levels of attentional lapses and demonstrated improved performance. These results, therefore, are inconsistent with the predictions of the resource-control account of sustained attention, as they showed that extended task duration in fact has positive effects on healthy older adults’ working memory performance.

Here, we posit that the occurrence of mind wandering should be also associated with fluctuations in activity in brain regions associated with executive control (the frontal-parietal and dorsal attention networks; Corbetta & Shulman, 2002; Corbetta et al., 2008; Posner & Dehaene, 1994; Vincent et al., 2008) and mind wandering (the default mode network; Raichle et al., 2001), and that these fluctuations should be inversely related (see also Esterman & Rothlein, 2019). According to this account, because normal aging is associated with significant decreases in the strength of functional connectivity density (i.e., the statistical relationship between brain regions; Tomasi & Volkow, 2010) in the dorsal attention and default mode networks (Tomasi & Volkow, 2012), the lower frequencies of mind wandering reported in healthy older adults could be attributable to less efficient switching and/or cooperation between these two networks to produce a train of thought during mind wandering (Smallwood et al., 2012). This proposal goes beyond the resource-control account of sustained attention, which cannot account for the findings that longer task duration does not lead to a higher incidence of mind wandering or more pronounced performance decrements in healthy older adults (Arnicane et al., 2021). In support of this proposal, studies have shown that mind wandering is associated with increased default mode network and decreased dorsal attention network activation (Christoff et al., 2009; Fortenbaugh et al., 2018; Kucyi et al., 2013; Mason et al., 2007; Robertson et al., 1997; Smallwood et al., 2013), indicating that there might be a push–pull relationship between these two networks that impacts the occurrence of mind wandering (cf. Esterman & Rothlein, 2019).

### Interim summary

We have shown that the switching perspective is a useful addition to the four prominent theories of mind wandering. While acknowledging that other factors may be at play, this newly formulated view not only provides a plausible explanation as to why healthy and cognitively impaired older adults experience a reduction in mind wandering, but it also provides new insights for determining the initiation of mind-wandering episodes. In the next section, we present evidence to support our view.

## Review of evidence supporting the switching perspective

The strongest evidence to date in support of this new perspective comes from research using the voluntary task**-**switching paradigm (Arrington & Logan, 2004), for which participants are free to switch tasks or continue with the same task at their preference. Somewhat paradoxically, research has consistently demonstrated that most of the participants decided to switch tasks despite negative consequences (i.e., switch costs; e.g., Irons & Leber, 2016; Kessler et al., 2009; Mittelstädt et al., 2019), although comparatively healthy older adults tended to initiate voluntary task switching less frequently than healthy young adults (Ardiale & Lemaire, 2012; Butler & Weywadt, 2013; Lockenhoff et al., 2020; Terry & Sliwinski, 2012). In light of cognitive aging, this finding may not seem surprising given that repeating the currently active task set requires fewer executive resources than switching to a different task set (Wirth et al., 2018) and switching between task sets or rules increases cognitive load (Arrington & Logan, 2004; Kool et al., 2010). In like manner, we argue that it should not be surprising either that healthy older adults and older adults with Alzheimer’s disease, Parkinson’s disease, and amnestic mild cognitive impairment experience less frequent mind wandering, as a reduced switching ability could contribute, at least in part, to getting “stuck” in a task**-**focus mode (cf. Walpola et al., 2020).

Additional evidence for the switching perspective of mind wandering comes from the intriguing finding that mind wandering does not always impair young adults’ performance in switching tasks, in contrast to tasks that require sustained attention. Using a probe**-**caught method, Kam & Handy, (2014) found no significant disruptive effects of mind wandering on task-switching performance. A further study, which investigated the association between mind wandering and task-switching performance over time, observed similar response times and accuracy rates for the trials leading up to “on-task” and “off-task” reports (Thomson et al., 2014), which again suggests that switching performance was unaffected by mind wandering. Similar results were obtained by Arnau et al., (2020), who investigated electrophysiological correlates of mind wandering during a switching task and did not observe slower response times during periods of self-reported mind wandering relative to on-task episodes.

The lack of performance costs, particularly response time costs, for switching tasks is puzzling because mind wandering has consistently been found to disrupt behavioral performance on tasks that tap the other two core executive function measures—inhibition (e.g., Kam & Handy, 2014; Smallwood et al., 2008; Stawarczyk et al., 2011) and working memory (e.g., Kam & Handy, 2014; Krimsky et al., 2017; Unsworth & Robison, 2016). Given this, one might expect that mind wandering should also significantly affect one’s task-switching performance. Although Kam & Handy, (2014) speculated that the null effect of mind wandering on switching-task performance might reflect cognitive flexibility being a less representative executive functioning skill (as it showed the weakest correlations with other executive function measures; for more details, see Miyake et al., 2000), these researchers also noted that switching from the task at hand to task-unrelated thoughts may be a form of switching. In the same manner, we posit that because switching either between task-related mental sets or between task-related and task-unrelated mental sets requires cognitive flexibility, when one mind wanders during performance of a switching task, they continue to engage in a “task-switching mind frame” (i.e., instead of switching between task-related mental sets, the individual switches between task-related and task-unrelated mental sets), and thereby can maintain task-switching performance. This conjecture appears to fit well with previous studies indicating that frequent task/response switches can shift the flexibility-persistence balance (Hommel, 2015) towards higher flexibility (e.g., Fröber & Dreisbach, 2017; Fröber et al., 2018; Zhuo et al., 2021; for a review, see Dreisbach & Fröber, 2019).

## Future directions

Acknowledging that instances of mind wandering are instances of mental set shifting (see Murray & Krasich, 2020, for a similar argument) opens up new avenues for future scientific investigations. First, to directly investigate this new perspective, future research could examine the association between cognitive flexibility and the tendency to mind wander, as despite a number of researchers using a task**-**switching paradigm as the primary task in their investigation of mind wandering (e.g., Arnau et al., 2020; Kam & Handy, 2014; Thomson et al., 2014), to our knowledge none have explicitly investigated the role of cognitive flexibility in mind wandering. Second, as there are many different types of switching (e.g., rule switching, task set switching, and response set switching), which have been found to activate different brain areas (Ravizza & Carter, 2008), it may be important to investigate how these switching abilities are related and which type is most closely associated with mind wandering. Determining this may help advance the current understanding of the higher incidence of mind wandering in ADHD, and may ultimately shed light on the inconsistent results regarding whether ADHD is associated with deficits in cognitive flexibility (e.g., Halleland et al., 2012; Irwin et al., 2019; Rohlf et al., 2012; Willcutt et al., 2005), which in turn may shed further light on the viability of the switching perspective forwarded here. Third, as an increasing number of studies have revealed distinct effects of intentional and unintentional mind wandering on task performance (e.g., Martínez-Pérez et al., 2021; Moran et al., 2021; Seli et al., 2016a, 2016b; for a review, see Seli, et al., 2016a, 2016b), future research could investigate whether intentional and unintentional mind wandering constitute distinct forms of task-set activation (e.g., counscious or uncounscious activation of task-unrelated mental sets; Arango-Muñoz & Bermúdez, 2021; Lau & Passingham, 2007; Reuss et al., 2011; Weibel et al., 2013) that involve distinct neural mechanisms and might differ with respect to their relationships with cognitive flexibility.

Fourth, considering that in a real-world setting we constantly multitask (e.g., writing an email while listening to music and eating a meal), and studies have found a positive association between self-reported frequency of concurrent use of multiple digital media streams and mind-wandering tendency (e.g., Kane et al., 2017; Ralph et al., 2014; Wiradhany & Koerts, 2021), future research could investigate the association between mind wandering, multitasking, and cognitive flexibility. Fifth, to understand past findings (e.g., McVay & Kane, 2009; Robison & Unsworth, 2018; Unsworth & Robison, 2020) in light of this switching perspective, future studies could investigate whether healthy young adults with higher self-reported mind-wandering tendencies have lower working memory capacity but superior switching abilities. In consideration of previous findings (e.g., McVay & Kane, 2009, 2012b; Miyake et al., 2000; Oberauer et al., 2003; Robison & Unsworth, 2018; Shipstead et al., 2015; Unsworth & Robison, 2020; as discussed in the “Limitations and an alternative viewpoint for the executive failure hypothesis”), the increased mind**-**wandering frequency seen in healthy young adults with lower working memory capacity could reflect a tendency to initiate more switches between task-related and task-unrelated mental sets in relation to superior switching abilities. Sixth, considering that the executive failure hypothesis (McVay & Kane, 2009, 2010, 2012b) fits with the data in healthy young adults and individuals with ADHD, but does not account for the data in older adult populations discussed in this review, future research should explore the association between cognitive flexibility and the tendency to mind wander in different populations with age in mind, as it could be the case that the executive failure hypothesis applies to young adults whereas the switching account of mind wandering applies to older populations.

## Concluding remarks

The findings reviewed in this article provide initial evidence to suggest that there may be an association between cognitive flexibility and mind wandering, and that distinct patterns of mind wandering may signal and be a product of altered cognitive flexibility. Although more research is needed, the switching perspective of mind wandering put forward here may provide a more comprehensive account of mind wandering that fits better with the experimental findings to date, including why healthy older adults and those with Alzheimer’s disease, Parkinson’s disease, and amnestic mild cognitive impairment experience less mind wandering (e.g., Gyurkovics et al., 2018; Jordão et al., 2019; Maillet & Schacter, 2016; Niedzwienska & Kvavilashvili, 2018; Walpola et al., 2020). This novel line of research may lead to the development of clinical detection tools and therapeutic approaches (e.g., task**-**switching training) aimed at preserving, or enhancing, the rates of mind wandering in populations with reduced levels of mind wandering (e.g., older adults), as mind wandering does have important functions such as facilitating future planning (e.g., Baird et al., 2011; Mazzoni, 2019; Seli, Maillet, et al., 2017; Seli, Ralph, et al., 2017; Stawarczyk et al., 2011) and the generation of creative ideas (e.g., Gable et al., 2019; Yamaoka & Yukawa, 2020) as well as promoting positive mood (e.g., Welz et al., 2018). Furthermore, recognizing that previous findings on mind wandering can be viewed from a switching perspective may provide an important contribution to our understanding of the basic psychological processes of mind wandering and its determinants, and may help future research to come up with a definition of mind wandering that will gain consensus in the field.

## Data Availability

Not applicable.
